# Bis(2,2′-bipyridine-κ^2^ *N*,*N*′)chloridocobalt(II) perchlorate

**DOI:** 10.1107/S1600536809049034

**Published:** 2009-11-25

**Authors:** Zhongjun Gao, Fahui Li

**Affiliations:** a Department of Chemistry, Jining University, Shandong 273155, People’s Republic of China; b Marine Drug and Food Institute, Ocean University of China, Qingdao 266003, People’s Republic of China

## Abstract

In the cation of the title compound, [CoCl(C_10_H_8_N_2_)_2_]ClO_4_, the Co^II^ atom displays a distorted trigonal-bipyramidal coordination geometry. The two pyridine rings in each 2,2′-bipyridine ligand form dihedral angles of 10.75 (12) and 4.28 (13)°. The crystal packing is stabilized by inter­ionic C—H⋯O hydrogen bonds, C—H⋯π inter­actions and aromatic π–π stacking inter­actions, with centroid–centroid distances of 3.616 (7) Å.

## Related literature

For the use of 2,2′-bipyridine in coordination chemistry, see: Ruiz-Perez *et al.* (2002 ▶). For the structure of the corresponding copper(II) compound, see: Harrison *et al.* (1981 ▶).
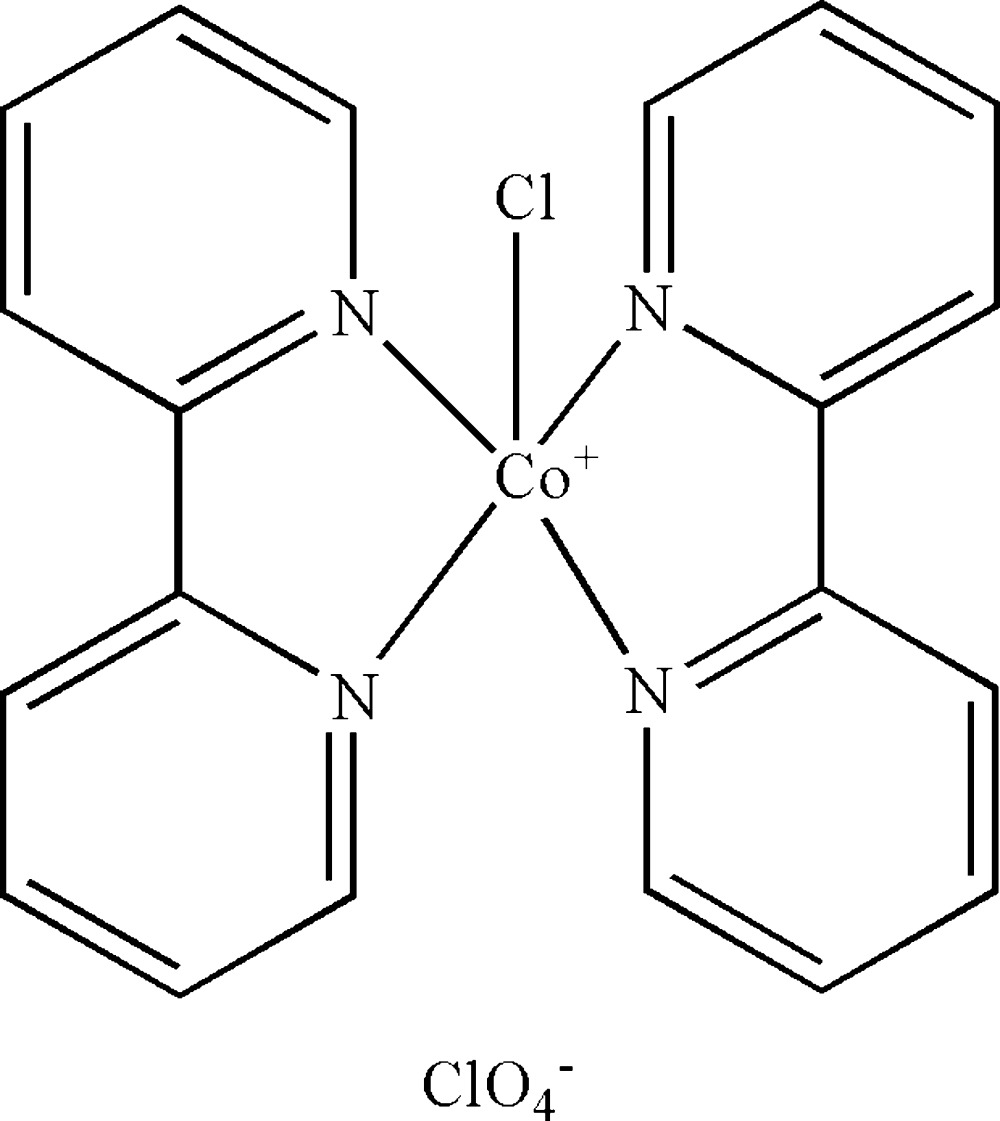


## Experimental

### 

#### Crystal data

[CoCl(C_10_H_8_N_2_)_2_]ClO_4_
*M* *_r_* = 506.20
Monoclinic, 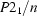

*a* = 10.7725 (12) Å
*b* = 12.2696 (14) Å
*c* = 16.333 (2) Å
β = 105.361 (2)°
*V* = 2081.7 (4) Å^3^
*Z* = 4
Mo *K*α radiation
μ = 1.12 mm^−1^
*T* = 298 K
0.40 × 0.21 × 0.19 mm

#### Data collection

Bruker SMART CCD diffractometer
Absorption correction: multi-scan (*SADABS*; Sheldrick, 1996 ▶) *T*_min_ = 0.664, *T*_max_ = 0.816
10284 measured reflections
3661 independent reflections
2458 reflections with *I* > 2σ(*I*)
*R*_int_ = 0.029

#### Refinement

*R*[*F* ^2^ > 2σ(*F* ^2^)] = 0.043
*wR*(*F* ^2^) = 0.124
*S* = 1.06
3661 reflections
280 parameters
1 restraint
H-atom parameters constrained
Δρ_max_ = 0.50 e Å^−3^
Δρ_min_ = −0.39 e Å^−3^

### 

Data collection: *SMART* (Bruker, 1998 ▶); cell refinement: *SAINT* (Bruker, 1998 ▶); data reduction: *SAINT*; program(s) used to solve structure: *SHELXS97* (Sheldrick, 2008 ▶); program(s) used to refine structure: *SHELXL97* (Sheldrick, 2008 ▶); molecular graphics: *SHELXTL* (Sheldrick, 2008 ▶); software used to prepare material for publication: *SHELXL97* and *PLATON* (Spek, 2009 ▶).

## Supplementary Material

Crystal structure: contains datablocks I, global. DOI: 10.1107/S1600536809049034/rz2393sup1.cif

Structure factors: contains datablocks I. DOI: 10.1107/S1600536809049034/rz2393Isup2.hkl

Additional supplementary materials:  crystallographic information; 3D view; checkCIF report

## Figures and Tables

**Table 1:** Selected bond lengths (Å)

Co1—N3	1.992 (3)
Co1—N1	1.992 (3)
Co1—N4	2.075 (4)
Co1—N2	2.138 (3)
Co1—Cl1	2.2645 (13)

**Table 2:** Hydrogen-bond geometry (Å, °)

*D*—H⋯*A*	*D*—H	H⋯*A*	*D*⋯*A*	*D*—H⋯*A*
C3—H3⋯O1^i^	0.93	2.41	3.170 (6)	139
C4—H4⋯O2^ii^	0.93	2.50	3.365 (6)	155
C10—H10⋯O3^iii^	0.93	2.53	3.116 (6)	122
C11—H11⋯*Cg*1^iv^	0.93	2.85	3.709 (6)	155

Symmetry codes: (i) 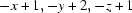; (ii) 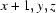; (iii) 
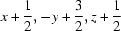
; (iv) 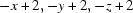. *Cg*1 is the centroid of the N2/C6–C10 pyridine ring.

**Fig. 1.**
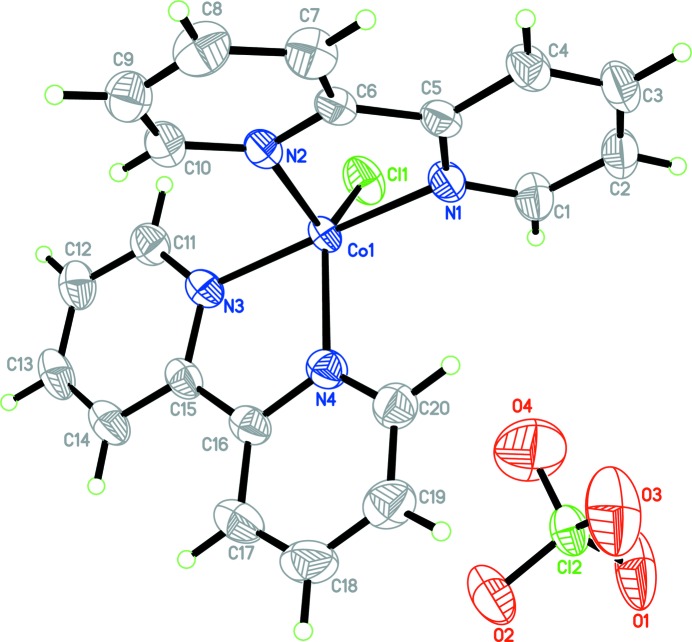
The molecular structure of the title compound with 30% displacement ellipsoids.

## supplementary crystallographic information

### Comment

Hydrogen bonding has been intensively investigated in organic crystalline solids, but is relatively unexplored in coordination complexes. In order to search the new functional hydrogen-bonded metal coordination network structures, chelating ligands such as 2,2'-bipyridine (Ruiz-Perez *et al.*, 2002) were selected for study because they can simultaneously coordinate with the metal ions and provide potential intermolecular interaction sites.

In title compound (Fig. 1), the cobalt(II) atom has a distorted trigonal bipyramidal coordination geometry provided by one chloride anion and four nitrogen atoms from the two chelating 2, 2'-bipyridine molecules. The equatorial plane is defined by the N2, N4 and Cl1 atoms, and the sum of the N—Co—N and N—Co—Cl angles is 360.0 (3)°. The apical positions are occupied by the N1 and N3 atoms [N1—Co1—N3 = 174.58 (14)°]. The Co—N bond lenghts (Table 1) lie in the range 1.992 (3)-2.138 (3) Å. The N1/C1–C5, N2/C6–C10 and N3/C11–C15, N4/C16–C20 pyridine rings form dihedral angles of 10.75 (12) and 4.28 (13)°, respectively. The structure is similar to that reported previously for the corresponding copper(II) compound (Harrison *et al.*, 1981). In the crystal structure, cations and anions interact through C—H···O hydrogen bonds (Table 2) to form a three-dimensional network. The structure is further stabilized by a C—H···π interaction (C11—H11···Cg1, 2.85 Å; C11—H11—Cg1, 155°; Cg1 is the centroid of the N2/C6–C10 pyridine ring) and by aromatic π–π stacking interactions involving centrosymmetrically related N3/C11–C15 pyridine rings, with a centroid-to-centroid distance of 3.616 (7) Å.

### Experimental

To a solution of Co(ClO_4_)_2_^.^6H_2_O and CoCl_2_^.^6H_2_O (1:1 molar ratio) in ethanol (10 mL) was added a solution of 2,2'-bipyridine (0.1562 g, 1 mmol) in ethanol (20 mL) and the resulting green solution was stirred for 8h at 333 K. The mixture was then filtered and the filtrate was allowed to stand at room temperature for one week to give well shaped green crystals suitable for X-ray analysis (yield 61%). Analysis calculated for C_20_H_16_Cl_2_N_4_O_4_Co: C 47.45, H, 3.19; N 11.07%; found: C 47.49, H 3.26, N, 11.12%.

### Refinement

All H atoms were positioned geometrically and constrained to ride on their parent atoms, with C—H = 0.93 Å and with *U*_iso_(H) = 1.2*U*_eq_(C).

### Crystal data

**Table:**

[CoCl(C_10_H_8_N_2_)_2_]ClO_4_	*F*(000) = 1028
*M**_r_* = 506.20	*D*_x_ = 1.615 Mg m^−^^3^
Monoclinic, *P*2_1_/*n*	Mo *K*α radiation, λ = 0.71073 Å
Hall symbol: -P 2yn	Cell parameters from 2895 reflections
*a* = 10.7725 (12) Å	θ = 2.6–25.8°
*b* = 12.2696 (14) Å	µ = 1.12 mm^−^^1^
*c* = 16.333 (2) Å	*T* = 298 K
β = 105.361 (2)°	Block, green
*V* = 2081.7 (4) Å^3^	0.40 × 0.21 × 0.19 mm
*Z* = 4	

### Data collection

**Table:**

Bruker SMART CCD diffractometer	3661 independent reflections
Radiation source: fine-focus sealed tube	2458 reflections with *I* > 2σ(*I*)
graphite	*R*_int_ = 0.029
φ and ω scans	θ_max_ = 25.0°, θ_min_ = 2.1°
Absorption correction: multi-scan (*SADABS*; Sheldrick, 1996)	*h* = −12→12
*T*_min_ = 0.664, *T*_max_ = 0.816	*k* = −14→14
10284 measured reflections	*l* = −12→19

### Refinement

**Table:**

Refinement on *F*^2^	Primary atom site location: structure-invariant direct methods
Least-squares matrix: full	Secondary atom site location: difference Fourier map
*R*[*F*^2^ > 2σ(*F*^2^)] = 0.043	Hydrogen site location: inferred from neighbouring sites
*wR*(*F*^2^) = 0.124	H-atom parameters constrained
*S* = 1.06	*w* = 1/[σ^2^(*F*_o_^2^) + (0.0432*P*)^2^ + 2.7771*P*] where *P* = (*F*_o_^2^ + 2*F*_c_^2^)/3
3661 reflections	(Δ/σ)_max_ = 0.001
280 parameters	Δρ_max_ = 0.50 e Å^−^^3^
1 restraint	Δρ_min_ = −0.39 e Å^−^^3^

### Special details

**Table:**

Geometry. All esds (except the esd in the dihedral angle between two l.s. planes) are estimated using the full covariance matrix. The cell esds are taken into account individually in the estimation of esds in distances, angles and torsion angles; correlations between esds in cell parameters are only used when they are defined by crystal symmetry. An approximate (isotropic) treatment of cell esds is used for estimating esds involving l.s. planes.
Refinement. Refinement of F^2^ against ALL reflections. The weighted R-factor wR and goodness of fit S are based on F^2^, conventional R-factors R are based on F, with F set to zero for negative F^2^. The threshold expression of F^2^ > 2sigma(F^2^) is used only for calculating R-factors(gt) etc. and is not relevant to the choice of reflections for refinement. R-factors based on F^2^ are statistically about twice as large as those based on F, and R- factors based on ALL data will be even larger.

### Fractional atomic coordinates and isotropic or equivalent isotropic displacement parameters (Å^2^)

**Table:**

	*x*	*y*	*z*	*U*_iso_*/*U*_eq_	
Co1	0.73259 (5)	0.95539 (4)	0.86267 (3)	0.04181 (18)	
N1	0.7739 (3)	0.9517 (3)	0.7508 (2)	0.0531 (9)	
Cl1	0.72296 (14)	1.13977 (10)	0.86111 (8)	0.0720 (4)	
Cl2	0.27386 (13)	0.89418 (10)	0.63357 (7)	0.0653 (3)	
N2	0.9032 (3)	0.8579 (3)	0.8932 (2)	0.0504 (9)	
N3	0.6929 (3)	0.9438 (3)	0.9747 (2)	0.0514 (9)	
N4	0.6035 (3)	0.8263 (3)	0.8383 (2)	0.0527 (9)	
O1	0.1665 (5)	0.9455 (4)	0.5784 (3)	0.140 (2)	
O2	0.2326 (4)	0.8325 (4)	0.6957 (2)	0.1056 (14)	
O3	0.3357 (5)	0.8273 (4)	0.5887 (3)	0.143 (2)	
O4	0.3567 (6)	0.9733 (4)	0.6800 (4)	0.163 (2)	
C1	0.7066 (5)	1.0065 (4)	0.6821 (3)	0.0656 (13)	
H1	0.6329	1.0440	0.6851	0.079*	
C2	0.7427 (5)	1.0089 (4)	0.6077 (3)	0.0682 (14)	
H2	0.6939	1.0468	0.5609	0.082*	
C3	0.8520 (5)	0.9544 (4)	0.6034 (3)	0.0691 (14)	
H3	0.8790	0.9562	0.5538	0.083*	
C4	0.9217 (5)	0.8970 (4)	0.6730 (3)	0.0617 (12)	
H4	0.9955	0.8593	0.6708	0.074*	
C5	0.8802 (4)	0.8963 (3)	0.7466 (3)	0.0485 (10)	
C6	0.9463 (4)	0.8365 (3)	0.8244 (3)	0.0472 (10)	
C7	1.0428 (5)	0.7611 (4)	0.8278 (3)	0.0658 (13)	
H7	1.0727	0.7473	0.7804	0.079*	
C8	1.0942 (5)	0.7065 (4)	0.9037 (4)	0.0742 (14)	
H8	1.1589	0.6552	0.9072	0.089*	
C9	1.0505 (5)	0.7275 (4)	0.9728 (3)	0.0674 (13)	
H9	1.0840	0.6909	1.0237	0.081*	
C10	0.9553 (4)	0.8044 (4)	0.9653 (3)	0.0624 (12)	
H10	0.9259	0.8198	1.0127	0.075*	
C11	0.7445 (5)	1.0087 (4)	1.0417 (3)	0.0623 (12)	
H11	0.8073	1.0588	1.0376	0.075*	
C12	0.7073 (5)	1.0034 (5)	1.1160 (3)	0.0689 (13)	
H12	0.7447	1.0487	1.1615	0.083*	
C13	0.6132 (5)	0.9294 (4)	1.1214 (3)	0.0711 (15)	
H13	0.5858	0.9245	1.1707	0.085*	
C14	0.5602 (5)	0.8629 (4)	1.0530 (3)	0.0679 (14)	
H14	0.4959	0.8135	1.0557	0.082*	
C15	0.6028 (4)	0.8700 (4)	0.9804 (3)	0.0526 (11)	
C16	0.5549 (4)	0.8017 (3)	0.9042 (3)	0.0526 (11)	
C17	0.4680 (5)	0.7166 (4)	0.8981 (4)	0.0720 (14)	
H17	0.4344	0.6999	0.9435	0.086*	
C18	0.4323 (5)	0.6573 (4)	0.8242 (4)	0.0835 (17)	
H18	0.3734	0.6006	0.8193	0.100*	
C19	0.4824 (5)	0.6808 (4)	0.7588 (4)	0.0751 (15)	
H19	0.4596	0.6404	0.7089	0.090*	
C20	0.5678 (5)	0.7659 (4)	0.7675 (3)	0.0632 (12)	
H20	0.6022	0.7823	0.7224	0.076*	

### Atomic displacement parameters (Å^2^)

**Table:**

	*U*^11^	*U*^22^	*U*^33^	*U*^12^	*U*^13^	*U*^23^
Co1	0.0476 (3)	0.0455 (3)	0.0378 (3)	−0.0002 (3)	0.0210 (2)	0.0038 (2)
N1	0.056 (2)	0.058 (2)	0.049 (2)	0.0005 (19)	0.0199 (17)	0.0079 (18)
Cl1	0.1044 (10)	0.0515 (6)	0.0683 (8)	−0.0031 (7)	0.0372 (7)	0.0040 (6)
Cl2	0.0797 (8)	0.0674 (8)	0.0556 (7)	0.0164 (7)	0.0302 (6)	0.0039 (6)
N2	0.050 (2)	0.057 (2)	0.048 (2)	−0.0015 (17)	0.0184 (17)	0.0080 (17)
N3	0.054 (2)	0.053 (2)	0.052 (2)	0.0006 (18)	0.0244 (17)	0.0030 (17)
N4	0.050 (2)	0.055 (2)	0.056 (2)	0.0011 (17)	0.0181 (17)	0.0032 (18)
O1	0.138 (4)	0.217 (6)	0.072 (3)	0.099 (4)	0.039 (3)	0.055 (3)
O2	0.107 (3)	0.131 (4)	0.093 (3)	0.021 (3)	0.051 (2)	0.047 (3)
O3	0.178 (5)	0.176 (5)	0.095 (3)	0.094 (4)	0.071 (3)	−0.006 (3)
O4	0.191 (6)	0.107 (4)	0.184 (6)	−0.048 (4)	0.034 (5)	−0.021 (4)
C1	0.073 (3)	0.072 (3)	0.052 (3)	0.011 (3)	0.018 (2)	0.010 (2)
C2	0.086 (4)	0.071 (3)	0.045 (3)	−0.004 (3)	0.013 (3)	0.007 (2)
C3	0.094 (4)	0.073 (3)	0.047 (3)	−0.013 (3)	0.031 (3)	−0.001 (3)
C4	0.073 (3)	0.066 (3)	0.055 (3)	−0.006 (3)	0.032 (2)	−0.009 (2)
C5	0.053 (3)	0.046 (2)	0.051 (2)	−0.012 (2)	0.022 (2)	−0.005 (2)
C6	0.049 (2)	0.045 (2)	0.051 (2)	−0.007 (2)	0.020 (2)	−0.0030 (19)
C7	0.070 (3)	0.061 (3)	0.071 (3)	0.006 (3)	0.026 (3)	−0.008 (3)
C8	0.068 (3)	0.061 (3)	0.090 (4)	0.015 (3)	0.014 (3)	−0.001 (3)
C9	0.067 (3)	0.064 (3)	0.070 (3)	0.007 (3)	0.016 (3)	0.011 (3)
C10	0.063 (3)	0.071 (3)	0.055 (3)	0.001 (3)	0.020 (2)	0.012 (2)
C11	0.069 (3)	0.065 (3)	0.057 (3)	0.000 (2)	0.023 (2)	−0.001 (2)
C12	0.085 (4)	0.075 (3)	0.051 (3)	0.014 (3)	0.026 (3)	0.004 (2)
C13	0.091 (4)	0.080 (4)	0.055 (3)	0.019 (3)	0.042 (3)	0.016 (3)
C14	0.072 (3)	0.073 (3)	0.071 (3)	0.008 (3)	0.040 (3)	0.020 (3)
C15	0.053 (3)	0.053 (3)	0.058 (3)	0.012 (2)	0.025 (2)	0.016 (2)
C16	0.048 (3)	0.047 (2)	0.067 (3)	0.008 (2)	0.022 (2)	0.015 (2)
C17	0.073 (3)	0.060 (3)	0.090 (4)	−0.004 (3)	0.034 (3)	0.016 (3)
C18	0.077 (4)	0.059 (3)	0.109 (5)	−0.019 (3)	0.016 (3)	0.007 (3)
C19	0.079 (4)	0.055 (3)	0.084 (4)	−0.006 (3)	0.007 (3)	−0.001 (3)
C20	0.065 (3)	0.063 (3)	0.060 (3)	−0.002 (2)	0.015 (2)	−0.001 (2)

### Geometric parameters (Å, °)

**Table:**

Co1—N3	1.992 (3)	C6—C7	1.381 (6)
Co1—N1	1.992 (3)	C7—C8	1.388 (7)
Co1—N4	2.075 (4)	C7—H7	0.9300
Co1—N2	2.138 (3)	C8—C9	1.358 (7)
Co1—Cl1	2.2645 (13)	C8—H8	0.9300
N1—C1	1.344 (6)	C9—C10	1.375 (6)
N1—C5	1.349 (5)	C9—H9	0.9300
Cl2—O3	1.383 (4)	C10—H10	0.9300
Cl2—O4	1.399 (5)	C11—C12	1.375 (6)
Cl2—O1	1.412 (4)	C11—H11	0.9300
Cl2—O2	1.428 (4)	C12—C13	1.381 (7)
N2—C10	1.335 (5)	C12—H12	0.9300
N2—C6	1.351 (5)	C13—C14	1.379 (7)
N3—C15	1.348 (5)	C13—H13	0.9300
N3—C11	1.349 (6)	C14—C15	1.382 (6)
N4—C20	1.342 (5)	C14—H14	0.9300
N4—C16	1.350 (5)	C15—C16	1.475 (6)
C1—C2	1.370 (6)	C16—C17	1.388 (6)
C1—H1	0.9300	C17—C18	1.374 (7)
C2—C3	1.372 (7)	C17—H17	0.9300
C2—H2	0.9300	C18—C19	1.351 (7)
C3—C4	1.379 (7)	C18—H18	0.9300
C3—H3	0.9300	C19—C20	1.373 (6)
C4—C5	1.388 (6)	C19—H19	0.9300
C4—H4	0.9300	C20—H20	0.9300
C5—C6	1.476 (6)		
			
N3—Co1—N1	174.58 (14)	N2—C6—C5	115.2 (4)
N3—Co1—N4	79.94 (14)	C7—C6—C5	123.6 (4)
N1—Co1—N4	96.21 (14)	C6—C7—C8	118.5 (5)
N3—Co1—N2	97.25 (13)	C6—C7—H7	120.8
N1—Co1—N2	79.29 (13)	C8—C7—H7	120.8
N4—Co1—N2	96.25 (14)	C9—C8—C7	120.5 (5)
N3—Co1—Cl1	93.53 (10)	C9—C8—H8	119.8
N1—Co1—Cl1	91.89 (11)	C7—C8—H8	119.8
N4—Co1—Cl1	137.20 (10)	C8—C9—C10	118.0 (5)
N2—Co1—Cl1	126.54 (10)	C8—C9—H9	121.0
C1—N1—C5	119.2 (4)	C10—C9—H9	121.0
C1—N1—Co1	123.5 (3)	N2—C10—C9	123.1 (4)
C5—N1—Co1	117.2 (3)	N2—C10—H10	118.5
O3—Cl2—O4	111.8 (4)	C9—C10—H10	118.5
O3—Cl2—O1	110.8 (3)	N3—C11—C12	122.2 (5)
O4—Cl2—O1	109.5 (4)	N3—C11—H11	118.9
O3—Cl2—O2	110.3 (3)	C12—C11—H11	118.9
O4—Cl2—O2	104.9 (3)	C11—C12—C13	118.6 (5)
O1—Cl2—O2	109.4 (3)	C11—C12—H12	120.7
C10—N2—C6	118.8 (4)	C13—C12—H12	120.7
C10—N2—Co1	128.0 (3)	C14—C13—C12	119.4 (4)
C6—N2—Co1	112.2 (3)	C14—C13—H13	120.3
C15—N3—C11	119.4 (4)	C12—C13—H13	120.3
C15—N3—Co1	116.4 (3)	C13—C14—C15	119.8 (5)
C11—N3—Co1	124.1 (3)	C13—C14—H14	120.1
C20—N4—C16	118.7 (4)	C15—C14—H14	120.1
C20—N4—Co1	127.7 (3)	N3—C15—C14	120.6 (4)
C16—N4—Co1	113.5 (3)	N3—C15—C16	114.8 (4)
N1—C1—C2	122.2 (5)	C14—C15—C16	124.5 (4)
N1—C1—H1	118.9	N4—C16—C17	120.5 (5)
C2—C1—H1	118.9	N4—C16—C15	115.0 (4)
C1—C2—C3	119.0 (5)	C17—C16—C15	124.5 (4)
C1—C2—H2	120.5	C18—C17—C16	119.3 (5)
C3—C2—H2	120.5	C18—C17—H17	120.4
C2—C3—C4	119.5 (4)	C16—C17—H17	120.4
C2—C3—H3	120.3	C19—C18—C17	120.2 (5)
C4—C3—H3	120.3	C19—C18—H18	119.9
C3—C4—C5	119.2 (5)	C17—C18—H18	119.9
C3—C4—H4	120.4	C18—C19—C20	118.5 (5)
C5—C4—H4	120.4	C18—C19—H19	120.7
N1—C5—C4	120.8 (4)	C20—C19—H19	120.7
N1—C5—C6	115.4 (3)	N4—C20—C19	122.7 (5)
C4—C5—C6	123.8 (4)	N4—C20—H20	118.6
N2—C6—C7	121.2 (4)	C19—C20—H20	118.6

### Hydrogen-bond geometry (Å, °)

**Table:**

*D*—H···*A*	*D*—H	H···*A*	*D*···*A*	*D*—H···*A*
C3—H3···O1^i^	0.93	2.41	3.170 (6)	139
C4—H4···O2^ii^	0.93	2.50	3.365 (6)	155
C10—H10···O3^iii^	0.93	2.53	3.116 (6)	122
C11—H11···Cg1^iv^	0.93	2.85	3.709 (6)	155

Symmetry codes: (i) −*x*+1, −*y*+2, −*z*+1; (ii) *x*+1, *y*, *z*; (iii) *x*+1/2, −*y*+3/2, *z*+1/2; (iv) −*x*+2, −*y*+2, −*z*+2.

## References

[bb1] Bruker (1998). *SMART* and *SAINT*. Bruker AXS Inc., Madison, Wisconsin, USA.

[bb2] Harrison, W. D., Kennedy, D. M., Ray, N. J., Sheahan, R. & Hathaway, B. J. (1981). *J. Chem. Soc. Dalton Trans.* pp. 1556–1565.

[bb3] Ruiz-Perez, C., Luis, P. A. L., Lloret, F. & Julve, M. (2002). *Inorg. Chim. Acta*, **336**, 131–136.

[bb4] Sheldrick, G. M. (1996). *SADABS*. University of Göttingen, Germany.

[bb5] Sheldrick, G. M. (2008). *Acta Cryst.* A**64**, 112–122. 10.1107/S0108767307043930 18156677

[bb6] Spek, A. L. (2009). *Acta Cryst.* D**65**, 148–155. 10.1107/S090744490804362X PMC2631630 19171970

